# Entomological inoculation rates in 2009 on Bioko Island, Equatorial Guinea

**DOI:** 10.1186/1475-2875-11-S1-P88

**Published:** 2012-10-15

**Authors:** Hans J Overgaard, Vamsi P Reddy, Simon Abaga, Abrahan Matias, Michael R Reddy, Vani Kulkarni, Christopher Schwabe, Luis Segura, Immo Kleinschmidt, Michel A Slotman

**Affiliations:** 1 Department of Mathematical Sciences and Technology, Norwegian University of Life Sciences, Ås, Norway; 2 Department of Entomology, Texas A&M University, College Station, TX, USA; 3 National Malaria Control Program, Ministry of Health, Malabo, Eguatorial Guinea; 4 Medical Care Development International Inc., Malabo, Eguatorial Guinea; 5 Department of Epidemiology and Public Health, Yale University, New Haven, CT, USA; 6 Swiss Tropical and Public Health Institute, Basel, Switzerland; 7 London School of Hygiene and Tropical Medicine, London, UK

## Background

The Bioko Island Malaria Control Project (BIMCP) in Equatorial Guinea started in 2004 with the goal to reduce malaria transmission and associated morbidity and mortality on Bioko Island. In 2009 the project was extended by a second 5-year term from 2009-2013. To achieve these goals a set of integrated interventions were implemented combining vector control, effective case management, improved management of malaria during pregnancy, public communication, monitoring and evaluation, and operational research. Vector control consists of indoor residual spraying (IRS) with bendiocarb and long-lasting insecticide treated bed-nets. Here we report the results from the human landing collections of 2009 on vector density, biting times, species composition, sporozoite rates, and entomological inoculation rates (EIR).

## Materials and methods

Regular adult overnight mosquito collections were carried out by the human landing method at three sites on the island.

## Results

In the northwestern Punta Europa area the outdoor and indoor EIRs were 1028 and 738, respectively (883 combined). In this area close to 100% of captured mosquitoes were *Anopheles gambiae* s.s. The outdoor and indoor EIRs in southeastern Riaba were 301 and 196, respectively (248 combined). Here species composition consisted of 46% *An. gambiae* s.s. and 54% *An. melas.* In southwestern Arena Blanca the indoor and outdoor EIRs were 162 and 127, respectively (145 combined) with almost 100% being *An. melas.*

## Conclusions

Large spatiotemporal variations in mosquito density, biting activity, species composition and sporozoite rates indicate that pockets of high malaria transmission remain despite a largely successful control program. These hotspots require additional specific targeted vector control interventions, e.g. larval control or environmental management.

